# Functional Imaging of *Rel* Expression in Inflammatory Processes Using Bioluminescence Imaging System in Transgenic Mice

**DOI:** 10.1371/journal.pone.0057632

**Published:** 2013-02-28

**Authors:** Xingyu Yang, Hua Jing, Kai Zhao, Ruilin Sun, Zhenze Liu, Yue Ying, Lei Ci, Ying Kuang, Fang Huang, Zhugang Wang, Jian Fei

**Affiliations:** 1 School of Life Science and Technology, Tongji University, Shanghai, China; 2 Shanghai Research Center for Model Organisms, Shanghai, China; 3 National Key Laboratory of Medical Neurobiology, Shanghai Medical College, Fudan University, Shanghai, China; Federal University of São Paulo, Brazil

## Abstract

c-Rel plays important roles in many inflammatory diseases. Revealing the dynamic expression of c-Rel in disease processes in vivo is critical for understanding c-Rel functions and for developing anti-inflammatory drugs. In this paper, a transgenic mouse line, B6-Tg(c-Rel-luc)^Mlit^, which incorporated the transgene firefly luciferase driven by a 14.5-kb fragment containing mouse c-Rel gene *Rel* promoter, was generated to monitor *Rel* expression in vivo. Luciferase expression could be tracked in living mice by the method of bioluminescence imaging in a variety of inflammatory processes, including LPS induced sepsis and EAE disease model. The luciferase expression in transgenic mice was comparable to the endogenous *Rel* expression and could be suppressed by administration of anti-inflammatory drug dexamethasone or aspirin. These results indicate that the B6-Tg(c-Rel-luc)^Mlit^ mouse is a valuable animal model to study *Rel* expression in physiological and pathological processes, and the effects of various drug treatments in vivo.

## Introduction

The transcription factor nuclear factor kappa B (NFκB) regulates a wide range of genes associated with cellular proliferation and inflammation [1]. There are five members in the NFκB family, RelA (p65), RelB, c-Rel, p50 and p52 [1]–[5]. The c-Rel, gene name is *Rel*, is mainly expressed in differentiated lymphoid and myeloid cells [4]. In unstimulated cells, c-Rel exists in cytoplasm as inactive homodimeric or heterodimeric complexes by binding an IκB protein (Inhibitor of κB). When cells are stimulated by certain cytokines, antigens, stress factors and viral or bacterial products, the IκB kinase (IKK) is activated and phosphorylates IκB. The phosphorylated IκB is degraded by the process of ubiquitination [1]–[3], [5]. The free homodimeric or heterodimeric c-Rel complexes then enter into the nucleus and bind to the κB sites of target genes to regulate their expression. The target genes of c-Rel are consisted of those controlling cell proliferation/cell growth, cell apoptosis/cell survival, cell adhesion/cell architecture, immune cell function and DNA repair/damage [3]–[5].

Upregualted expression of *Rel* was detected in macrophages following lipopolysaccharide (LPS) stimulation [6]. Increased *Rel* expression had also been reported in breast cancer [7]. It was reported that *Rel* knockout mice showed less severity in the development of inflammatory bowel disease, asthma, type I diabetes, acute destructive arthritis, liver fibrosis, experimental autoimmune encephalomyelitis (EAE) [8], cardiac hypertrophy and fibrosis [1], [9]–[14]. Patients, with negative c-Rel expression, suffering the Diffuse Large B-cell Lymphoma with a germinal center B-cell phenotype, would have better overall survival [15], while the present of c-Rel was critical to the survival in a cecal ligation and puncture (CLP) mouse model of sepsis [16]. However, the mechanisms of *Rel* and its dynamic changes in these physiological and pathological processes are largely unknown. In this paper, we have established a transgenic mouse model by using the mouse *Rel* promoter to direct the expression of luciferase reporter gene. A highly sensitive, efficient, and non-invasive small–animal imaging system was adopted for capturing the photons produced by the luciferase in real time to trace *Rel* expression in living animals [17], [18]. Our data demonstrated that this model can be successfully used to study the transcriptional regulation of *Rel* expression in various inflammatory disease processes and evaluate the effects of anti-inflammatory drugs in vivo.

## Results

### Generation and Molecular Characterization of B6-Tg(c-Rel-luc)^Mlit^ Transgenic Mouse

**Figure 1:**
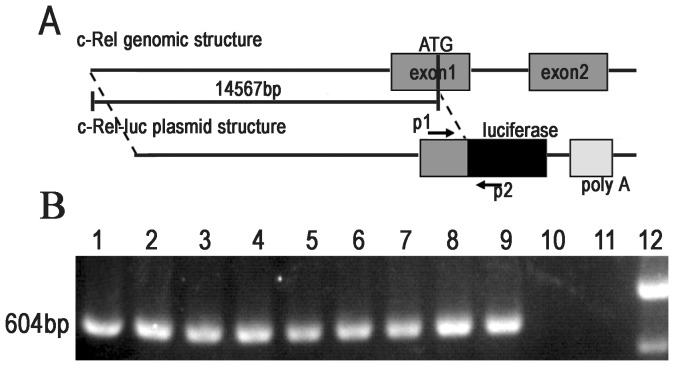
Schematic diagram of pc-Rel-luc reporter construct and identification of the transgenic founders. (A) The c-Rel-luc transgene harboring a 14.6 kb mouse *Rel* gene promoter and firefly luciferase cDNA was diagramed. Primer P1 and P2 was indicated. P1: forward-luc primer, P2: reverse-luc primer. (B) A 604-bp fragment was amplified by PCR with primer P1 and P2 in transgenic founder mice. PCR products were separated on 1% agarose gel. Lane 1–9: the products from transgenic founders; lane 10: from a wild-type C57BL/6 control; lane 11: from ddH_2_O; lane 12: DNA marker DL 2,000 ladder.

The schematic diagram of c-Rel-luc transgene is showed in Fig. 1A. The 14567 bp DNA fragment upstream from the translational initial codon ATG of mouse *Rel* gene was used as the promoter to drive the expression of firefly luciferase. The PCR primers designed for identification of the transgene were located in the region of *Rel* sequence and the luciferase gene (Fig. 1A). Nine founders were obtained (Fig. 1B) and the transgenic mice, here named as B6-Tg(c-Rel-luc)^Mlit^, were backcrossed to wild type C57BL/6J mice at least for five generations for further studies.

### Expression of Luciferase in B6-Tg(c-Rel-luc)Mlit Mice Induced by LPS or Zymosan

**Figure 2:**
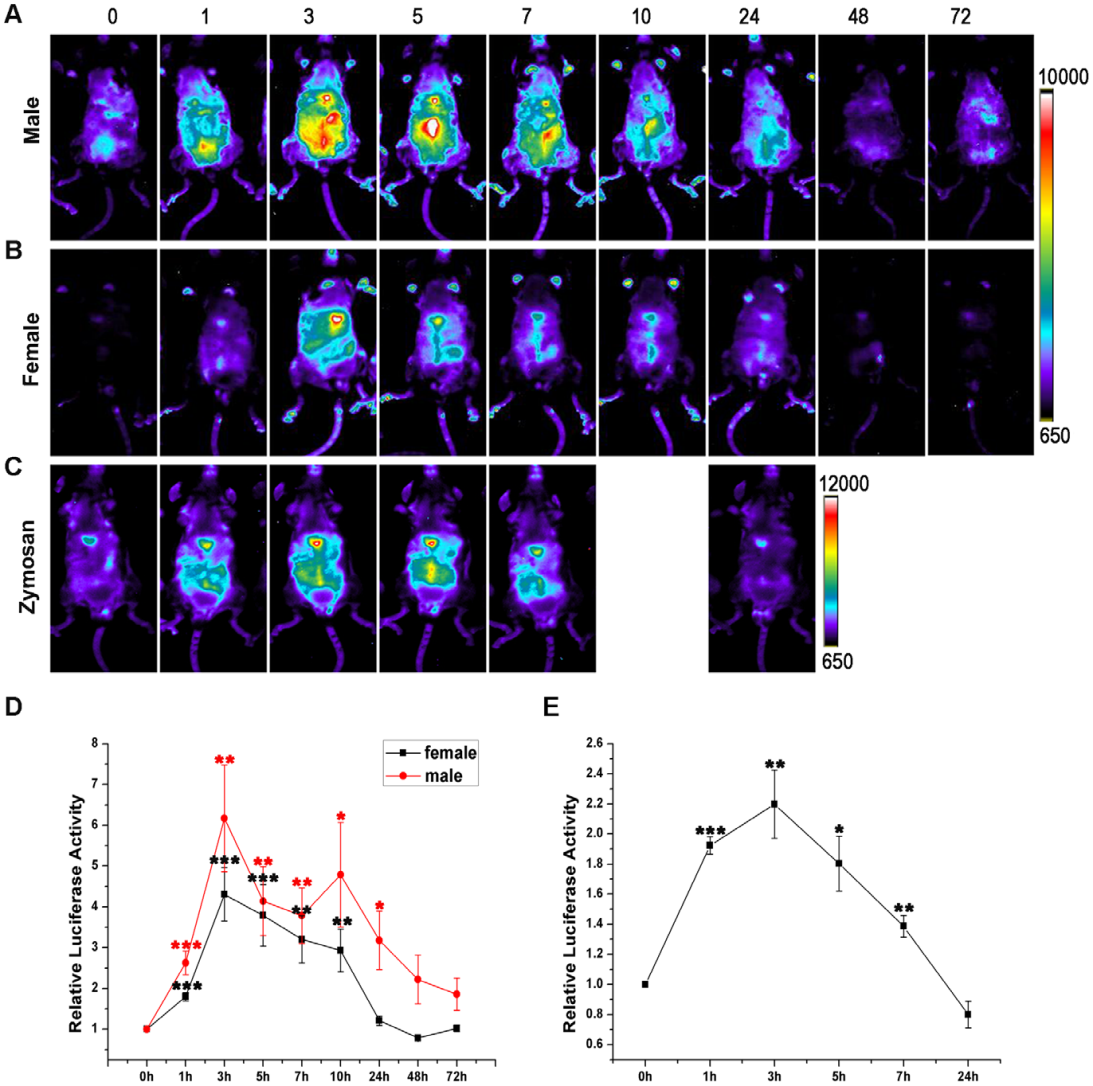
Induction of luciferase expression in B6-Tg(c-Rel-luc)^8Mlit^ mice stimulated by LPS or Zymosan. The ventral representative images were depicted for male (A) and female (B) mice treated with LPS. (C) Male B6-Tg(c-Rel-luc)^8Mlit^ mice were treated with zymosan (5 mg/kg). The color scale in intensity/sec was shown at the right. (D) The relative LPS induced luciferase activity. The changes of LPS induced luciferase activity were shown in fold, n = 7. (E) The changes of zymosan induced luciferase activity in fold, n = 3 in each group. *p<0.05; **p<0.01; ***p<0.001.

The offspring of each founder were injected intraperitoneally with LPS to test the responses of luciferase expression. Four of the nine founders showed a significant induced luciferase activity across the body after LPS i.p. injection. The line derived from the founder 8, B6-Tg(c-Rel-luc)^8Mlit^, with the lowest baseline of luciferase activity and highest LPS-inducible luciferase expression was chosen for the further studies. The expression of luciferase could be detected at 1 hour after LPS injection in B6-Tg(c-Rel-luc)^8Mlit^ mice. The luminescent signal peaked at 3 h post injection in both females and males compared to the base line luminescence. In male mice, the signal peak was as much as 6.5 fold of the base line, while the value is 4.5 fold in females (n = 7 per group, *p*<0.002) (Fig. 2A, B). Then the signals gradually declined and returned to the base line by 48 h (n = 7 per group, *p*>0.05). Both male and female mice had a similar tendency of LPS-induced luminescent signal changes. The time course of luciferase activities after LPS treatment were present in Fig. 2D, E.

Zymosan, a cell wall particle from *Saccharomyces cerevisiae*, could also induce luciferase expression in the B6-Tg(c-Rel-luc)^8Mlit^ mice (Fig. 2C, E) which was similar to LPS treatment. The luciferase activity peak was at 3 hour after injection compared to the base line measured at the start time point (n = 3 per group, *p*<0.01).

### Comparison of Luciferase Activity Profile and Endogenous Rel mRNA Expression Pattern in LPS Treated B6-Tg(c-Rel-luc)8Mlit Mice

**Figure 3:**
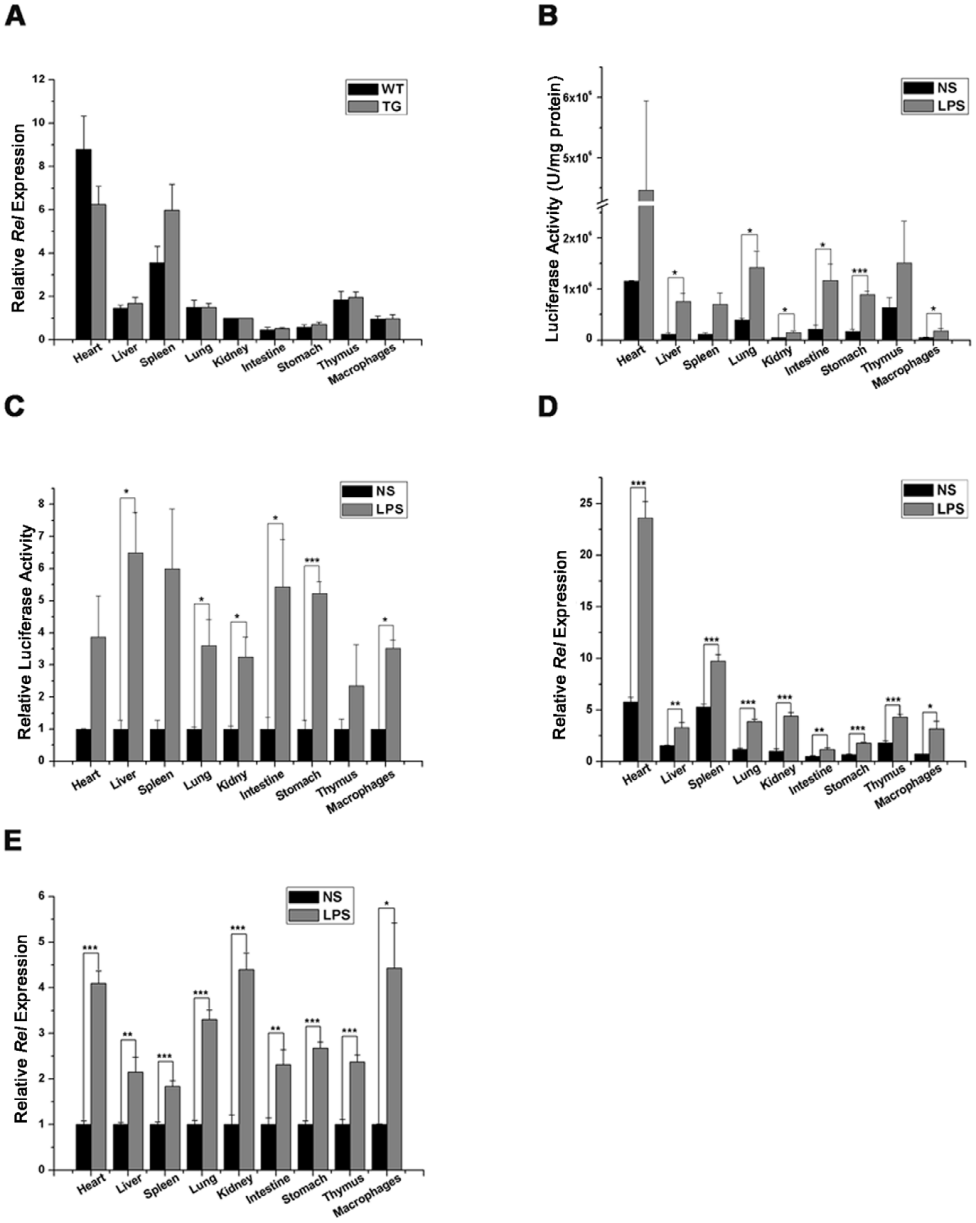
Comparison of luciferase acitivity profile and endogenous *Rel* mRNA expression pattern in B6-Tg(c-Rel-luc)^8Mlit^ mice following LPS treament. (A) The *Rel* expression were analyzed by RT-PCR in the B6-Tg(c-Rel-luc)^8Mlit^ mice (TG) and wild type littermates (WT) without LPS treatment, n = 3 in each group. (B)The luciferase activity in selected organs was measured in male B6-Tg(c-Rel-luc)^8Mlit^ mice at 3 hour after either LPS (3 mg/kg) or saline (NS) treatment. n = 3 in each group. (C) The ratio of LPS induced luciferase activity in each group to that of saline treated control (NS), n = 3. (D) The *Rel* gene expression in selected organs was measured by RT-PCR in the male transgenic mice at 3 hour after LPS or saline treatment (NS). The *Rel* mRNA level in saline treated kidney was set as 1, in each group, n = 3. (E) The ratio of LPS induced endogenous *Rel* expression in each group to that of saline treated control (NS). n = 3, *p<0.05; **p<0.01; ***p<0.001.

The *Rel* expression in B6-Tg(c-Rel-luc)^8Mlit^ mice was comparable with that of wild type littermates (Fig. 3A). This result indicated that the transgene and the process of transgenic processing did not modify the endogenous *Rel* expression in mouse. The ex vivo measurement of LPS induced luciferase activity and endogenous *Rel* mRNA expression in B6-Tg(c-Rel-luc)^8Mlit^ mice were performed. The mouse organs were dissected and homogenated at the time point of 3 h after LPS injection. The luciferase activity was high in the heart, liver, spleen, intestine and stomach. Compared with the saline treated mice, luciferase activity in LPS treated group could be increased by 6.5 fold in the liver, 6 fold in the spleen, 5.2 fold in the intestine, 5 fold in the stomach, 4 fold in the heart, 3.8 fold in the lung, 3 fold in the kidney, 2.3 fold in thymus. In addition, the luciferase activity reached to 3.2 fold in the macrophages (Fig. 3B, C). Meanwhile, the endogenous *Rel* expression was quantified by real time PCR (Fig. 3D, E). Compared with the saline group, LPS stimulated the endogenous *Rel* expression by 2.1 fold in the liver, 1.8 fold in the spleen, 2.3 fold in the intestine, 2.6 fold in the stomach, 4.1. fold in the heart, 3.3 fold in the lung, 4.4 fold in the kidney, 2.3 fold in the thymus and 4.4 fold in macrophages (Fig. 3D, E). These results showed that the luciferase activity profile and its response to LPS treatment were in parallel with that of endogenous *Rel* mRNA expression in B6-Tg(c-Rel-luc)^8Mlit^ mice.

### Inhibition of LPS Induced Luciferase Activity in B6-Tg(c-Rel-luc)8Mlit Mice by Dexamethasone or Aspirin

**Figure 4:**
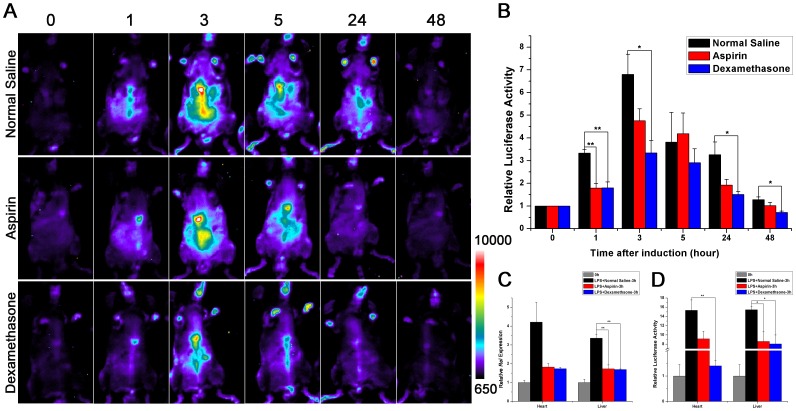
Inhibition of LPS induced luciferase activity in B6-Tg(c-Rel-luc)^8Mlit^ mice by dexamethasone or aspirin. (A) The LPS-induced luciferase activity in B6-Tg(c-Rel-luc)^8Mlit^ mice was inhibited both by aspirin (5 mg/kg) and dexamethasone (3 mg/kg) treatment. The mice were imaged at 0, 1, 3, 5, 24, 48 hour post injection, n = 3 (A) and the quantification of luciferase activity (intensity/sec) was presented in (B); The *Rel* expression in the heart and liver of B6-Tg(c-Rel-luc)^8Mlit^ mice were measured ex vivo after LPS injection with or without aspirin or dexamethasone co-treatment, n = 3. The mRNA expression level (C) and the luciferase activity (D) were presented. n = 3. *p<0.05; **p<0.01.

Dexamethasone and aspirin, the commonly used anti-inflammatory drugs, are reported inhibiting LPS induced sepsis [19]. In our study, the LPS-induced luciferase activity in B6-Tg(c-Rel-luc)^8Mlit^ mice could be significantly inhibited by the drug treatment (Fig. 4A). The luciferase signal were 6.8 fold as the baseline at 3 hour after LPS injection, while the values were only 3.3 fold and 4.7 fold in dexamethasone or aspirin co-treated group, respectively. The luciferase signals in the dexamethasone or aspirin co-treated group were declined to the baseline at 24 hours after LPS injection, while the signals in the control group took about 48 hours to return to the baseline (Fig. 4B). Comparing with the LPS/normal saline double treated transgenic mouse, *Rel* mRNA expression and luciferase activity were significantly lower in heart and liver of the LPS/dexamethasone or aspirin double treated mice at 3 hours after i.p. injection (Fig. 4C, D). These data indicated that B6-Tg(c-Rel-luc)^8Mlit^ mice could be used as a sensitive and reliable model to evaluate anti-inflammatory drugs in vivo.

### Luciferase Expression in B6-Tg(c-Rel-luc)8Mlit Mice of Myelin Oligodendrocyte Glycoprotein35–55 (MOG) Induced Experimental Autoimmune Encephalomyelitis (EAE) Model

**Figure 5:**
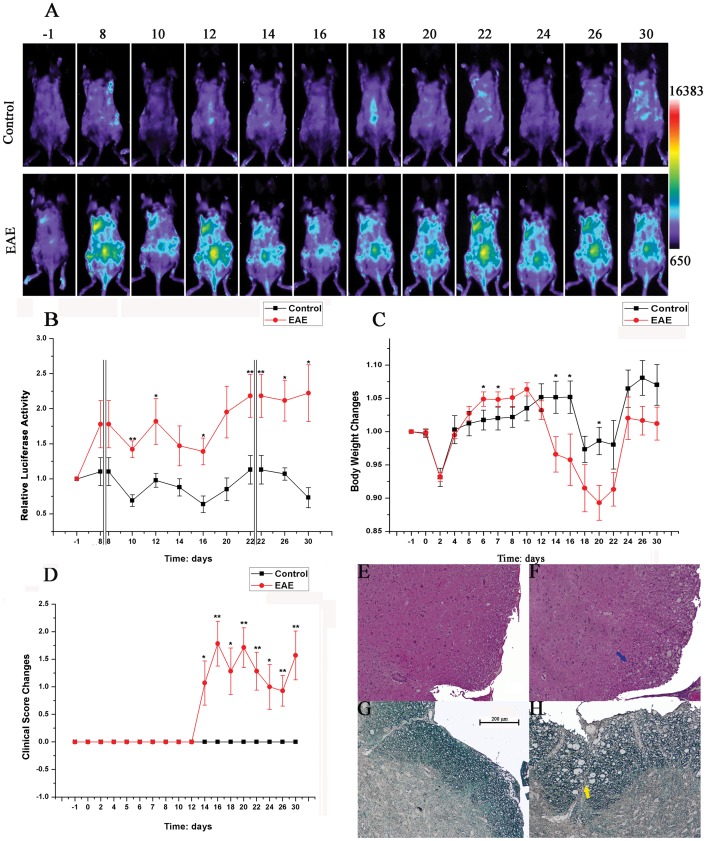
Luciferase expression in B6-Tg(c-Rel-luc)^8Mlit^ mice of MOG induced experimental autoimmune encephalomyelitis (EAE) model. (A) Female B6-Tg(c-Rel-luc)^8Mlit^ mice were treated with CFA and with or without emulsionized MOG. Images were captured at day −1, 8, 10, 12, 14, 16, 18, 20, 22, 24, 26 and 30; (B) The quantification of luciferase activity; (C) The changes in body weight of EAE and control mice; (D) The clinical scores of EAE and control mice, n = 7; (E, F) Mouse spinal cord sections stained with H&E. (E) Section from the control mouse; (F) Section from the EAE mouse. The infiltration of inflammatory leukocytes was indicated by the blue arrow. (G, H) Mouse spinal cord sections stained with LFB. (G) Section from the control mouse; (H) Section from the EAE mouse. The demyelination in the white matter was marked by the yellow arrow. *p<0.05; **p<0.01.

To validate if B6-Tg(c-Rel-luc)^8Mlit^ mice could be used in the research of chronic inflammatory disease, we studied the dynamic change of luciferase expression in EAE model. Compared with the control group, the luciferase signal was observed at the dorsum area of EAE mice, from day 8 after MOG challenge, and then was maintained high and waved during the whole disease process (Fig. 5A). Compared with the control group, the EAE group’s body weight reduced sharply starting at day 12 when the clinical symptom emerged (Fig. 5C). The development of the disease was confirmed by hematoxylin & eosin staining (HE) and luxol fast blue (LFB) staining at day 16, 4 days after the disease onset and near the peak of clinical activity. The HE-stained spinal cord sections were presented in Fig. 5E, F. The results exhibited massive infiltration of inflammatory leukocytes in the EAE group. Accompanied by the inflammatory leukocytes accumulation, the demyelination in the white matter was obvious by LFB staining (Fig. 5G, H). It is notable that the change of luciferase signal intensity (Fig. 5B) was much earlier than that of clinical score (Fig. 5D), which suggested that the luciferase activity of the transgenic mouse model could be an indicator to determine the initial development and severity of EAE disease.

## Discussion

We have established a *Rel* report transgenic mouse model which can be used to monitor the endogenous *Rel* expression. Using in vivo bioluminescence imaging detection system, we demonstrated that luciferase activity in B6-Tg(c-Rel-luc)^8Mlit^ mice was dramatically induced after i.p injection of LPS. The result of ex vivo experiment showed that the luciferase expression was induced in the heart, liver, spleen, lung, kidney, intestine, stomach, thymus and macrophages after the treatment with LPS, especially in the heart, liver, spleen, intestine and stomach. The data were in consistent with the change of endogenous murine *Rel* mRNA expression induced by LPS treatment. Meanwhile, the patterns of luciferase expression in LPS-treated B6-Tg(c-Rel-luc)^8Mlit^ mice were also comparable to the results of *Rel* expression reported previously [20]. The fold change did not match exactly between the luciferase activity and endogenous *Rel* mRNA expression. However, this is understandable that the protein level is often not in linear correlation with the endogenous mRNA expression in cells [17]. Dexamethasone and aspirin, two well-known anti-inflammatory drugs [19], [21]–[23], suppressed the induction of luciferase expression and endogenous *Rel* expression in LPS treated B6-Tg(c-Rel-luc)^8Mlit^ mice. These data demonstrate that the transgenic mice are faithful for monitoring *Rel* expression in vivo in c-Rel involved physiological or pathological processes and for evaluating the effects of anti-inflammatory drugs. LPS could induce *Rel* expression in monocytes and macrophages [24]. We also collected macrophages from the abdomen fluid of the transgenic mouse. These specific cells manifested a good response of luciferase expression to LPS stimulus in vitro and could be used for high-throughput screening and studying of anti-inflammatory drugs at cellular level (data not show).

Zymosan, a cell wall particle derived from *Saccharomyces cerevisiae,* is also widely used to induce inflammation in various relative experiments [25]. However, the receptors for zymosan are distinct from those for LPS. LPS, the part of the outer cell wall of Gram negative bacteria, is detected by TLR4 [26], [27], while zymosan is recognized by TLR2 and TLR6. Although LPS and zymosan activates different triggers of target cells, the following inflammatory cascades seem to be similar [25]. Our data also support the conclusion, for both LPS and zymosan could induce similar luciferase expression profiles in the B6-Tg(c-Rel-luc)^8Mlit^ mice.

The mouse EAE model is routinely used to study molecular mechanisms and signaling pathways of inflammatory regulation in Multiple sclerosis (MS) [28]. It was reported that c-Rel-deficient mouse was resistant to the development of EAE due to its defective in the IL-12 and IFN-γ induction and in the Th1 responses [5], [8]. It suggested that *Rel* expression was involved in the disease process of EAE. In our experiments, the luciferase expression in EAE group increased significantly at the eighth day after MOG injection before the loss of body weight and clinical symptoms occurred, which indicated that *Rel* up-regulation played an important role in the onset of EAE. Furthermore, the dynamic changes in the bioluminescence intensity of luciferase activity are related to clinical score and disease location. Our result showed at the first time the dynamic and spatial expression patterns of *Rel* in EAE mouse model and provided a distinctive mouse model to study EAE development and related drug screening.

The *Rel* gene in human being is a susceptibility locus in a variety of immune diseases, including rheumatoid arthritis, celiac disease, psoriasis, ulcerative colitis, primary sclerosing cholangitis, and B cell lymphoma [3]. So the B6-Tg (c-Rel-luc)^8Mlit^ mice can be used to trace *Rel* expression in these pathological processes and study the effects of relative drug treatments.

## Materials and Methods

### Ethics Statement

All animals were housed in specific pathogen-free environment. Animal handling was performed in accordance with institutional guidelines and approved by the Institutional Animal Care and Use Committee in Shanghai Research Center for Model Organisms and the IACUC permit number was 20110004. When the experiments were finished, the mice were euthanized with CO_2_.

### Reagents

Bacterial LPS, zymosan, dexamethasone, aspirin and complete Freund adjuvant (CFA) were purchased from Sigma-Aldrich (St. Louis, MO, US). Potassium Luciferin (Gold Biotechnology, St. Louis, MO, US) was dissolved in PBS (Beyotime, China) at 15 mg/ml and stored at −20°C. Pertussis toxin was purchased from Calbiochem (Germany).

### Construction of c-Rel-luc Vector and Generation of Transgenic Mice

The luciferase coding region and poly A signal was cut from the PGL3-Basic (Clontech, Mountain View, CA, US) by the restriction enzyme BamH I and Kpn I. The fragment was inserted into a modified pBR322 and the recombinant plasmid was named as pBR322-luc. A DNA fragment containing 14567 bp upstream sequence from translation initiation codon ATG of the *Rel* gene was retrieved from BAC clone BMQ53G1 (Bacpac Resources Children’s Hospital Oakland, CA, US) into pBR322-luc by ET cloning strategy to direct the expression of luciferase gene. The final plasmid, named as pc-Rel-luc, was verified by BamH I and Hind III digestion and DNA sequencing. The plasmid pc-Rel-luc was linearized and served as transgene cassette to produce transgenic mice in the C57BL/6J background by standard microinjection techniques. The transgenic mice were bred to C57BL/6J for five generations before testing.

Transgenic founders and their offspring were identified by PCR. The forward primer p1 is 5′ TCGGGGGTGGGAAGGTGTGA3′ and the reverse primer p2 is 5′ GGCGCAACTGCAACTCCGATAAA 3′. The position of p1 and p2 were indicated in Fig. 1A.

### In vivo Imaging of Luciferase Activity

In vivo imaging was performed as previously described using a lumazone imaging system (Mag Biosystems, Tucson, AZ, US) [17]. The mice were shaved and injected intraperitoneally with 150 mg/kg luciferin (dissolved in PBS, PH = 7.4). Mice were anesthetized with the mixture of isoflurane/oxygen and then placed on the imaging stage. 12 minutes after luciferin injection, mice were imaged in the completely darkness room for 3 minutes. Photons emitted from specific regions were collected by the Lumazone and were quantified using Lumazone Version 2.0 software. The luciferase activity was presented in photon intensity per second.

### Screening of Luciferase Expression Transgenic Lines

For primary screening of transgenic lines, three luciferase gene positive mice of each gender from different transgenic founders were injected by LPS and imaged for the expression of luciferase transgene at 0 and 3 h post injection. The LPS dosage used was 3.0 mg/kg body weight. The transgenic lines with lowest baseline luciferase activity and highest inducible luciferase expression were selected for further study.

### Inducing Luciferase Expression in the Transgenic Mice by Intraperitoneal Injection of LPS or Zymosan

Acute septic shock model was induced by i.p. injection of LPS (3.0 mg/kg) or zymosan (5.0 mg/kg) into transgenic mice at the age of 2–3 months. Control mice were treated with normal saline. The luciferase activity was monitored through imaging at 0 h, 1 h, 3 h, 5 h, 7 h, 10 h, 24 h, 48 h and 72 h post injection. At the selected time points after the injection, mice were i.p. injected with the luciferin and imaged 12 minutes later with the lumazone imaging system as described above. To test the anti-inflammatory effects of dexamethasone and aspirin on LPS-induced luciferase expression, mice were co-treated with LPS and dexamethasone or aspirin. The control group was injected with LPS and normal saline. The mice were kept in a warm place with jelly inside the cage after i.p. injection. The luciferase activity was monitored through imaging at 0, 1, 3, 5, 24, 48 hour post injection. The mice were anaesthetized with sodium pentobarbital (0.08 mg/kg body weight) and sacrified at 0 h and 3 h post injection to detect the *Rel* mRNA and luciferase activity changes in heart and liver.

### Total RNA Isolation and Real Time PCR

Male transgenic mice, 2–3 month of age, were treated with i.p. injection of LPS (3.0 mg/kg), and the control ones were injected with normal saline. After 3 hours, total RNA was isolated from selected mouse tissues using Trizol (Tiangen, China) and the extracted RNA were kept at −80°C before using. Male transgenic mice, 5–6 month of age, were treated with LPS and co-treated with normal saline or dexamethasone or aspirin for 3 hours, and total RNA were isolated from heart and liver for further studying.

800 ng RNA samples from LPS or normal saline-treated transgenic mice were reverse transcribed into cDNA using Quant Reverse Transcriptase (Tiangen, China). For real time PCR amplification, 1 µL cDNA and 0.1 µM primer sets were used in the SuperReal SYBR Green Premix (Tiangen, China). Real time PCR was performed on the Realplex (Eppendorf, Germany). The reaction conditions were as follows: 95°C/4 min; 40 cycles of 94°C/10 s, 60°C/20 s, 72°C/30 s; 95°C/15 s, 60°C/15 s. The murine β-actin was used as a reference to normalize the *Rel* gene expression levels. The experiments were performed in triplicate. Data was obtained as threshold cycle (CT) values. Fold changes relative to normal saline controls were determined by the 2^−△△CT^ method.

The primers for real time PCR were synthesized by Invitrogen, Shanghai, China and listed below: Forward murine *Rel*: 5′ AGCTGCTGGACATTGAAGAC 3′, Reverse murine *Rel* : 5′ CAGAAATATTTCATCTCCTCC3′. Forwad murine *β-actin* : 5′ CCTGTATGCCTCTGGTCGTA3′, Reverse murine *β-actin* : 5′ CCATCTCCTGCTCGAAGTCT3′.

### Ex vivo Measurement of Luciferase Activity in the Transgenic Mouse Tissues

About 50–100 mg mouse tissues were dissected and lysed with 500 µL passive lysis buffer (Promega, Wisconsin, US). After centrifugation at 12 000 rpm for 10 minutes at 4°C, the supernatant was collected. Luciferase activity was measured using a Luminometer (Lumat LB9507, EG&G, Berthold, Germany) and the Luciferase Assay System (Promega). Protein concentration was estimated by Bradford Reagent (Bio-Rad, CA, US).

### Macrophages Harvest

Mice were anaesthetized with sodium pentobarbital (0.08 mg/kg body weight) and sacrificed by dislocation of vertebrae cervicales, soaked in 75% alcohol for 5 minutes, then i.p. injected with 5 ml PBS and the abdomen fluid was collected. The cells were collected by centrifugation at 1000 g for 5 min and treated with 1 ml Red Blood Lysis Buffer (Beyotime, China) for 1 min. After centrifugation at 5000 g for 5 min, the supernatant was discarded and the macrophages were cultured in RPMI 1640 (Takara, Japan) at 37°C. After 2 hours, the cells were washed using PBS for two times and cultured with RPMI 1640 medium containing 10% FBS, penicillin and streptomycin for another 48 hours before luciferase activity assay.

### Experimental Autoimmune Encephalomyelitis (EAE)

Female mouse, 6–8 weeks old, were subcutaneously immunized with emulsionized 300 µg myelin oligodendrocyte glycoprotein_35–55_ (MOG, MEVGWYRSPFSRVVHLYRNGK, GL Biochem, China) dissolved in 100 µL CFA and 100 µL PBS. On the day of the immunization and 48 h later, each mouse was i.p. injected with 200 ng Pertussis toxin. The control mice were treated by the same reagents except that the MOG was excluded. MOG immunized mice and controls were examined for disease symptoms at certain time points. They were scored for monitoring disease severity on the basis of the following scale: 0, no clinical signs; 1, limp tail; 2, paraparesis (weakness, incomplete paralysis of one or two hind limbs); 3, paraplegia (complete paralysis of two hind limbs); 4, paraplegia with forelimb weakness or paralysis; 5, moribund or death [29], [30]. Selected animals were killed at the time of peak symptoms, the spinal cord were removed and fixed in 10% paraformaldehyde. Paraffin-embedded 5 µm sections of the spinal cord were stained with hematoxylin and eosin and Luxol fast blue (Genmed Scientifics INC, China). When the experimental mice started to show the clinical symptom, they were placed in warm and with jelly in their cages to provide the water and energy. The litter was changed every three days. When the mice were scored 5, they were euthanized with CO_2_. We never observed any mouse reached to score 5 in this project.

### Statistics

All data were expressed as means ± SE. The data were analyzed by one-way ANOVA through Origin 8.0. P value of less than 0.05 was considered significant.
